# Exploring the balance between functionality and aesthetics: an analytical framework and pragmatic consideration of the anthropomorphism of service robots

**DOI:** 10.3389/fpsyg.2025.1555395

**Published:** 2025-04-02

**Authors:** Xin Xin, Wenchao Liu

**Affiliations:** ^1^Business School, Jilin Business and Technology College, Changchun, China; ^2^School of Business Administration, Jilin University of Finance and Economics, Changchun, China

**Keywords:** service robot, anthropomorphism, appearance anthropomorphism, behavior anthropomorphism, psychological mechanisms

## Abstract

The phenomenon of anthropomorphism in service robots has gained significant traction across multiple service industries; however, there remains a lack of a robust theoretical framework that adequately elucidates the preference for anthropomorphic design. Following the systematic review methodology, our findings indicate that the design of service robots necessitates a careful equilibrium between functional capabilities and aesthetic considerations. Central to the concept of anthropomorphism in service robots is the attribution of human-like characteristics, motivations, intentions, and emotions to their perceived or actual behaviors, aimed at enhancing human comprehension of robotic actions and promoting meaningful social interactions. From a design standpoint, anthropomorphism fulfills cognitive requirements while also serving as a mechanism for inductive reasoning. Influential factors in the anthropomorphism of service robots include the activation of human subject knowledge, effectiveness motivation, and social motivation, alongside additional variables such as individual personality traits, contextual elements, developmental phases, and cultural backgrounds, all of which exert a direct impact. The anthropomorphism of service robots has engendered significant considerations regarding human expectations, the perceived intelligence of robots, and the management of anthropomorphic attributes. A thorough investigation into the mechanisms that underpin the anthropomorphic interpretation of service robots, along with the practical implications that emerge, can assist various service organizations in determining the appropriate types and levels of anthropomorphic service robots, while also facilitating customers’ engagement with these robots in a more rational and contextually appropriate manner.

## 1 Introduction

The proliferation of service robots across diverse sectors, including healthcare (encompassing companionship, elder care, surgical assistance, rehabilitation, and hospital sanitation), retail (such as customer greeting and beverage service), hospitality and travel (including information provision at transportation hubs and hotel navigation), and food and beverage services (like ordering, payment processing, and food delivery) (Mende et al., 2019), has prompted a growing emphasis on optimizing human-robot interactions. Consequently, an increasing number of service robots are being designed with anthropomorphic features, leading to the phenomenon of “anthropomorphism” in robot design. Anthropomorphism refers to the inclination to attribute human-like traits, motivations, intentions, or emotions to the actual or perceived behaviors of nonhuman entities (Epley et al., 2007). A plausible hypothesis posits that individuals are inclined to engage with machines in a manner akin to interpersonal communication (Fong et al., 2003). The anthropomorphism of robots has emerged as a significant area of inquiry within the domain of human-computer interaction, garnering considerable interest from fields such as robotics and marketing in recent years (Mende et al., 2019; Kim et al., 2016; van Pinxteren et al., 2019). Empirical studies indicate that both the aesthetic and functional attributes of robots substantially influence human perceptions, interaction behaviors, and the propensity to form enduring relationships with these entities (Bartneck and Forlizzi, 2004). By integrating human-like characteristics, employing facial expressions and other social signals, and replicating human communication methods (including language, eye contact, and gestures) into robot design, the level of anthropomorphism can be significantly enhanced. The implementation of anthropomorphic design elements and features reflective of “human society” can foster greater familiarity with robots, elicit social responses, and consequently improve user acceptance (Hur et al., 2015; Kim et al., 2016; Duffy, 2003; Fink, 2012).

The function of anthropomorphism in robotics is not primarily to replicate human beings, but rather to serve as a means of enhancing interaction between humans (Duffy, 2002). Furthermore, an overreliance on anthropomorphic designs, particularly in the case of humanoid robots that closely resemble humans yet remain artificial constructs, may elicit adverse reactions, including fear or aversion (Mori et al., 2012). The degree of a robot’s resemblance to human characteristics enhances its familiarity; however, there exists a threshold beyond which minor discrepancies in appearance and behavior elicit a sense of unease. This phenomenon is referred to as the “Uncanny valley” (Mac Dorman and Ishiguro, 2006; Mori et al., 2012). Consequently, any deviations in the robot’s behavior from the normative human expectations held by users may result in feelings of fear or discomfort. Empirical studies indicate that humanoid robots are more likely to provoke reluctance and negative responses from individuals compared to robots designed with pet-like or more utilitarian forms (Austermann et al., 2010). In essence, the objective of anthropomorphism in robotics is to create systems that can effectively operate within our physical and social environments, thereby facilitating social interaction (Duffy, 2003). Consequently, it is imperative to strike a balance between functionality and aesthetic form, as the latter supports the former. The anatomical and functional characteristics of humans should not necessarily serve as the definitive model for robotic design, given that robots are fundamentally machines rather than human entities; designs that closely mimic human attributes may constrain the potential capabilities of these machines. The concept of anthropomorphism in robotics is inherently intricate, yet it intuitively endows robots with significant physical and social attributes, which are anticipated to be increasingly utilized in forthcoming research on social robotics. Nevertheless, there remains a lack of theoretical frameworks within the academic literature that elucidate the reasons behind the prevalent adoption of anthropomorphic designs in service robots and the perceptions of customers regarding these anthropomorphic robotic systems.

Is there an optimal anthropomorphism approach within the domain of service robots? What cognitive mechanisms contribute to humans’ propensity to anthropomorphize service robots? Which facets of human characteristics should be prioritized to augment and improve the social functionalities of robots? Under what conditions might the anthropomorphism of robots surpass the thresholds of human acceptability? A thorough investigation and analysis of these questions will aid in dispelling prevalent misconceptions in human-computer interaction, highlight the essential components of anthropomorphism, and strive for a harmonious alignment between societal expectations and the capabilities of machines, ultimately leading to the development of effective solutions for social robots.

## 2 Service robot design: achieving a balance between functionality and aesthetics

Louis Sullivan, a seminal figure in modernist design, articulated a fundamental principle of modernist design in 1896: “Form always follows function, which is the universal law of all things” (Crabbe, 2013). This principle posits a linear causal relationship in the design process, wherein the identification of necessary functionalities precedes the determination of the appropriate form to realize those functionalities (Crabbe, 2013). Sullivan’s assertion has significantly influenced numerous generations of designers. However, subsequent scholars have observed that Sullivan’s maxim appears to be more relevant to the domain of engineering design, where the notion of function is predominantly confined to physical utility, neglecting the interplay between utility and communicative function (Crabbe, 2013).

The relationship between the form and function of a product is not characterized by opposition or isolation (Townsend et al., 2013). Within the engineering domain, product functionality and technical performance are frequently viewed as fundamental components, whereas in fields such as industrial design, which prioritize user experience and aesthetics, the significance of form tends to prevail (Townsend et al., 2011). Product functionality encompasses the specifications and standard architecture of a product, fundamentally representing the practical aspects of product design (Townsend et al., 2011). This functionality reflects the instrumental and practical characteristics of a product in fulfilling specific tasks or objectives (Dai et al., 2024). Functional design is comprised of a variety of factors, benefits, features, and attributes that contribute to utility (Talke et al., 2009), and it pertains to the extent to which products and services perform as anticipated (Batra and Ahtola, 1991). The functional attributes of a product enhance perceived utility, illustrating how design can facilitate more effective and comfortable usage by consumers (Hertenstein et al., 2005). Consequently, product functionality should incorporate design elements that address consumer needs and related concerns (Townsend et al., 2011). Conversely, the form of a product is defined by its structural features, which provide the framework for achieving functional attributes and embody the hedonic aspects of design (Townsend et al., 2011). This form pertains to the visual appearance or design that elicits sensory or aesthetic pleasure (Dai et al., 2024). By accentuating or concealing various technological aspects, form can shape consumer perceptions, offer visual cues that activate different cognitive modes, assist consumers in comprehending products, and evoke sensory experiences that influence cognition and emotion (Rindova and Petkova, 2007). Therefore, in the product design process, it is imperative to consider not only the practicality of product functions but also the aesthetic qualities of product form.

Product design decisions are generally made collaboratively or in a sequential manner by a multidisciplinary team comprising industrial designers, engineers, and marketing professionals. Each of these functional departments brings distinct perspectives regarding the critical elements of design. Industrial designers primarily emphasize the aesthetic aspects of products, whereas engineers prioritize functional features, viewing them as non-negotiable components. The role of industrial designers is to establish the external form of a product, ensuring aesthetic coherence that enables users to engage with the product’s functionality. Conversely, engineers are tasked with formulating technical specifications that govern the manufacturing process, product performance, and overall form. Marketing personnel adopt a demand-side perspective, focusing on consumer perceptions and preferences concerning various product functions, features, and attributes. While product design decisions are often influenced by these functional divergences and other organizational constraints, it is imperative that the final decision is informed by consumer feedback. A critical responsibility of a product development manager is to comprehend the trade-offs that must be navigated throughout the production process (Townsend et al., 2013). The product design process typically necessitates a balance among product functionality, structure, and aesthetics (Berkowitz, 1987). Berkowitz elucidates that the functional dimension of a product encompasses the needs and interests associated with its selection and preparation, while the structural characteristics are a reflection of these functional attributes. Bloch (1995) asserts that a product’s form serves as the basis for psychological responses, which are largely influenced by the goals and constraints imposed by the environment and the functions related to the product. Furthermore, the anticipated performance of a product and the utility it delivers are not mutually exclusive; rather, they are interconnected through functional limitations that may emerge during the design process (Townsend et al., 2011).

In the process of designing the shape and structure of robots, it is essential to consider a multitude of factors comprehensively. Primarily, the aesthetic design of the robot should align with its intended function. When robots are developed to perform specific tasks for human users, it is imperative that their design reflects a degree of “product specificity” to foster user comfort during operation. Additionally, in contexts where peer interaction is significant, robots must exhibit a certain level of “humanity” to facilitate comfortable social interactions with users (Fong et al., 2003). Consequently, the design of service robots should strive to achieve a harmonious balance between functionality and aesthetic form. Currently, the prevailing design trend for service robots involves the integration of anthropomorphic elements with social robot technology. This design philosophy is predicated on the understanding that both the appearance and functionality of a product can significantly influence human perception, interaction, and the potential for establishing enduring relationships with the technology (Bartneck and Forlizzi, 2004). Robots that incorporate human-like design features are capable of eliciting social responses from humans, thereby enhancing their acceptance (Duffy, 2003). In comparison to designs that prioritize functionality alone, individuals tend to respond more favorably to artificial entities that display humanoid characteristics, such as emotions and facial expressions. However, it is important to note that user preferences are often contingent upon specific tasks and contextual factors (Goetz et al., 2003). Therefore, the visual design of robots should be congruent with their capabilities and the expectations of users. The personification of technological agents appears to foster a form of social connection, which aids individuals in learning to utilize the technology and engaging with it in a positive manner (Epley et al., 2007). Users are generally more inclined to collaborate with robots that are capable of exhibiting social responses.

The propensity for individuals to attribute human-like characteristics to artificial products, including service robots, can be examined through two primary hermeneutic frameworks (Lee et al., 2005). The first perspective pertains to the design of artificial products, wherein anthropomorphism is perceived as a straightforward human reaction to life-like or social cues emitted by objects or systems, necessitating minimal psychological processing. Individuals frequently employ stereotypes and heuristic reasoning when interacting with objects or systems that exhibit signals reminiscent of life or social interaction. In the realm of design, the visual attributes of an object significantly influence overall perception (Schmitz, 2011). If this assertion is accurate, it follows that individuals may instinctively respond to the social cues presented by robots, thereby applying established patterns and norms of human social interaction to their engagements with these machines (Lee et al., 2005).

The second perspective is grounded in a human-centered cognitive approach. Individuals comprehend the functionality of artificial products through specific psychological models that inform their understanding of these systems’ operational mechanisms. When a system’s behavior mirrors that of humans--such as producing human-like sounds--individuals may align their psychological models of the system’s behavior with their models of human behavior, albeit with potential discrepancies in critical aspects (Lee et al., 2005). Furthermore, individuals’ assessments of robots’ “knowledge” and capabilities significantly influence their relationships with these entities. Prior research has substantiated the relevance of mental models across various types of robots, revealing that individuals possess more elaborate mental models for anthropomorphic robots compared to mechanical ones (Lee et al., 2005; Kiesler and Goetz, 2002).

## 3 The connotation and fundamental motivation of anthropomorphism

### 3.1 The connotation of anthropomorphism

Anthropomorphism has been recognized as a complex phenomenon that has eluded comprehensive explanation for centuries. Philosophers like Hume (1757/1957) has linked anthropomorphism to human frailty, positing that it represents a form of human folly that can only be mitigated through diligent education and critical thought. Some theorists even argue that anthropomorphism is an intrinsic aspect of human nature, suggesting that individuals have a propensity to perceive non-human entities as akin to themselves (Epley, 2018). The term “anthropomorphism” is derived from the Greek words “anthropos” meaning “person” and “morph” meaning “form” (Duffy, 2002), and it describes the inclination to attribute human traits to non-human subjects. These subjects may include non-human animals, natural forces, deities, and mechanical or electronic devices (Epley et al., 2007). According to the Oxford Dictionary, anthropomorphism refers to the attribution of human characteristics or behaviors to deities, animals, or inanimate objects (Soanes and Stevenson, 2005). Consequently, the essence of anthropomorphism lies in the imbuing of non-human entities--whether imagined or real--with human-like characteristics, motivations, intentions, and emotions (Epley et al., 2007; Epley, 2018).

Anthropomorphism, a related concept, involves inferring unobservable attributes of non-human subjects rather than merely describing their observable or imagined behaviors (Epley et al., 2007). This process extends beyond the animistic attribution of life to inanimate objects; it encompasses the transcendence of mere behavioral descriptions to include imaginative or observable actions (such as the affectionate behavior of dogs) and the application of human-like descriptors to convey the mental or physical traits of agents (for instance, describing dogs as self-loving). Thus, the core of anthropomorphism resides in the attribution of human-like qualities, behavioral traits, appearances, emotions, or mental states to both real and imagined non-human agents and objects (Epley et al., 2007; Leyens et al., 2003). Additionally, the conscious experiences, metacognitive abilities, and intentional features associated with human perception constitute fundamental components of anthropomorphism (Gray et al., 2007).

The practice of attributing human characteristics to inanimate objects, animals, and other entities serves to facilitate our understanding and interpretation of their behaviors. Personification involves ascribing cognitive or emotional states to these entities based on observational data, thereby rendering their actions more comprehensible within a particular social context. Humans exhibit a prevalent inclination to anthropomorphize objects, which entails merging the actual or perceived behaviors of non-human agents with human traits, motivations, intentions, or emotions. This anthropomorphic tendency can significantly influence interpersonal interactions with these entities (Epley et al., 2007). In contemporary society, individuals frequently engage with a variety of inanimate objects, including computers, automobiles, and mobile devices (Reeves and Nass, 1996). Despite the fact that these objects are devoid of consciousness, consumers often attribute human-like psychological attributes such as intentions, beliefs, attitudes, and knowledge to them. This process of personification is a widespread phenomenon within the realm of human social cognition (Epley, 2018).

### 3.2 The underlying motivations for anthropomorphism

From a psychological standpoint, anthropomorphism can be understood as the process of employing inductive reasoning to ascribe human-like characteristics to non-human entities, including service robots (Epley et al., 2007). The fundamental cognitive operations involved in this reasoning process are largely analogous to those found in other forms of inductive reasoning. These operations encompass the acquisition of knowledge, the activation of pre-existing knowledge, and the application of this activated knowledge toward specific objectives (Higgins, 1996). Individuals frequently engage in the anthropomorphism of non-human entities, which is primarily driven by three core motivations: the pursuit of social connections, motivational influences, and perceived similarities.

Firstly, the pursuit of social connections represents a fundamental human necessity (Baumeister and Mark, 1995). Social connection motivation encapsulates the individual’s intrinsic desire to forge social connections with others. The act of anthropomorphism fulfills this need by fostering a perceived relationship between human and non-human entities. As the most socially inclined primate species, humans derive greater levels of happiness and wellbeing from interpersonal interactions (Cacioppo and William, 2008). Specifically, in instances where individuals experience a deficiency in social connections, they are inclined to anthropomorphize non-human objects, such as robots, thereby addressing their social motivation. This suggests that a lack of social connection enhances the tendency toward anthropomorphism, while a strong sense of social connection diminishes it. Consequently, the motivation to forge social bonds with various non-human agents, such as deities or companion animals, may enhance cognitive focus on these entities, thereby increasing the likelihood of attributing human-like characteristics to them. Empirical studies provide support for this perspective, demonstrating instances of anthropomorphism in relation to non-human animals and religious figures (Epley et al., 2008; Bartz et al., 2016), as well as in the context of consumer products (Mourey et al., 2017; Chen et al., 2018).

The second motivation for this inquiry is the aspiration to elucidate, forecast, and potentially exert control over agents exhibiting humanoid traits. This motivation is frequently identified as “effect motivation” within the realm of psychological literature (White, 1959). Effectiveness motivation pertains to the individual’s inherent need to engage with their environment in a productive manner (White, 1959). Within the framework of anthropomorphism, effectiveness is manifested in the capacity for meaningful interactions between individuals and non-human agents, such as service robots. This capacity also encompasses the enhancement of individuals’ comprehension of complex stimuli and their ability to anticipate future behaviors of these agents. It is posited that, possibly as a result of evolutionary pressures associated with cohabitation in large social groups (Dunbar, 1993), humans have developed a distinctive and intricate social cognitive framework that enables them to interpret and anticipate the behaviors of others (Herrmann et al., 2007), commonly referred to as “theory of mind.” Humans do not perceive others merely as objects; rather, they attribute thoughts to them, encompassing intentions, desires, attitudes, and beliefs that serve to elucidate their actions. The concept of “mind” is employed by individuals to rationalize the behavior of nearly any autonomous agent, whether it be a human being or a geometric figure exhibiting independent or interdependent movement (Scholl and Patrice, 2000). When there is a need to account for an agent’s behavior, irrespective of its human or non-human status, the observer’s theory of mind is likely to be activated. By attributing human traits and motivations to service robots, individuals can improve their understanding of the robots’ actions, mitigate uncertainties associated with them, and bolster their confidence in predicting future behaviors. A series of experiments has demonstrated that when the behavior of a device is unpredictable, individuals are more inclined to ascribe it to humanoid cognition, thereby necessitating an explanation (Waytz et al., 2010). Consequently, anthropomorphism may be influenced by the same motivational mechanisms that drive our consideration of the thoughts of others.

The third motivation is derived from the parallels between observable characteristics of individuals and human perception, a phenomenon commonly referred to as “induced individual knowledge” (Epley, 2018). The phenomenon of anthropomorphism is fundamentally rooted in human self-awareness, which serves as a framework for interpreting the characteristics of unfamiliar entities. A critical factor influencing anthropomorphism is the activation of self-referential knowledge, as posited by Epley et al. (2007), which pertains to individuals’ understanding of their own nature. This self-knowledge is often more comprehensive and developed than the knowledge individuals possess regarding non-human entities. The previously mentioned desire for social connections and motivational influences are both top-down processes that facilitate the exchange of ideas among individuals and non-human entities. In contrast, perceived similarity operates as a bottom-up process that emanates from the agent itself, reflecting the similarities among humans. This perceived similarity encompasses various attributes, including facial features, movements, and vocalizations (Morewedge et al., 2007; Schroeder et al., 2017). Given the associative nature of the human brain, these attributes are likely to activate concepts associated with humanoid cognition. Consequently, when individuals engage in evaluative judgments, they are predisposed to draw upon knowledge associated with human traits or attributes, while information pertaining to non-human entities is typically acquired through more complex cognitive processes. In the context of anthropomorphizing non-human factors, both self-knowledge and the knowledge structures that have been acquired are simultaneously activated, thereby influencing the application of this knowledge toward specific objectives. This mechanism illustrates a cognitive process that enhances the activation of self-referential knowledge (Epley et al., 2007). For instance, in an experimental context, when an autonomous vehicle is ascribed a gender, is capable of communicating with users, and can anticipate its surroundings, its degree of anthropomorphism is significantly enhanced, as these traits suggest the presence of humanoid cognitive processes (Waytz and Michael, 2014).

## 4 The practical realization of anthropomorphism in service robots

According to Webster’s Dictionary, a robot is defined as “any mechanical organism that performs tasks in a manner that appears to be human through automated mechanical devices, especially remote control” (Duffy, 2003). In contemporary contexts, fostering interaction between humans and robots is regarded as a primary impetus for the anthropomorphic design of robotic systems. To facilitate meaningful social interactions with humans, robots must exhibit a certain level of anthropomorphic traits, whether in their form, behavior, or both (Duffy, 2003). The concept of anthropomorphism serves as a critical variable in the design of service robots; developers frequently employ anthropomorphism techniques, such as incorporating human facial features, enabling human-like limb movements, producing human vocalizations, and assigning names to robots rather than utilizing model numbers, to enhance their interactions with employees and customers in social settings (Zeller and David, 2014). For instance, Hanson Robotics Ltd. has named its most advanced humanoid robot Sophia, which evokes a perception of being “alive,” “newborn” and possessing “human emotions” (Mende et al., 2019). The emphasis on anthropomorphism aims to highlight human characteristics to foster trust, attachment, and a willingness to engage with these robotic entities.

In the design of social interactive robots, anthropomorphic elements are crucial, primarily manifested in the robot’s form (appearance) and behavior (including movement and interaction) (Dubois-Sage et al., 2023). Research indicates that individuals tend to ascribe human-like attributes to robots, with these attributions influenced by a variety of factors. These factors encompass not only characteristics inherent to the robots themselves but also situational elements pertaining to the context of interaction, as well as human factors related to the user (Dubois-Sage et al., 2023). The field of robotics leverages these anthropomorphic characteristics to enhance the acceptance of robots and facilitate interactions between humans and machines (Fong et al., 2003; Fink, 2012; Kim et al., 2019). The extent of anthropomorphism in a robot’s appearance pertains to how closely the robot resembles a human in its physical characteristics. Walters et al. (2008) categorized service robots into three distinct types based on their appearance’s anthropomorphic degree: Mechanoid, Humanoid, and Android. This classification has gained considerable recognition within the field (Belanche et al., 2019). Conversely, the anthropomorphism of robot behavior refers to the extent to which robots mimic human actions, including aspects such as movements, language, and emotional expressions. Kim et al. (2019) further delineated the anthropomorphism of robot behavior into two distinct levels: high anthropomorphic state and low anthropomorphic state. In a high anthropomorphic behavior state, service robots exhibit complex limb movements, nodding, changes in body posture, variations in vocal volume, and emotional expression in speech. In contrast, low anthropomorphic behavior states are characterized by a limited range of body movements and emotional expressions. The specific details of these classifications are presented in Table 1.

**Table 1:** Primary expressions of anthropomorphism in service robots.

Form	Connotation	Classification	Definition
Appearance anthropomorphism	The resemblance between robots and humans in terms of physical appearance is primarily evident in their anthropomorphic characteristics, including the presence of facial features and bodily structures.	Mechanoid	Robots that exhibit a predominantly mechanical appearance are characterized by a minimal presence of human-like features and a low level of anthropomorphism, commonly designated as mechanical bodies (Walters et al., 2008; Belanche et al., 2019).
		Humanoid	Although these robots may not closely resemble humans in their overall appearance, they exhibit certain human-like characteristics, including partial or complete representations of the head, face, eyes, ears, eyebrows, arms, hands, and legs. Their mobility can be facilitated through wheels or bipedal locomotion. The extent of anthropomorphism in their design is considered to be moderate (Walters et al., 2008; Belanche et al., 2019).
		Android	This robot is designed to closely mimic human appearance through advanced technological means. Its design incorporates highly anthropomorphic characteristics (Walters et al., 2008; Belanche et al., 2019).
Behavior anthropomorphism	Robots convey emotions through their physical movements, vocalizations, and linguistic expressions during interactions, demonstrating behavioral traits that resemble those of humans.	Highly anthropomorphic state	Service robots possess the capability to manipulate their limbs, exhibit nodding behavior, modify their body posture, and adjust their vocal volume, thereby facilitating the expression of emotions during verbal interactions (Kim et al., 2019).
		Low anthropomorphic state	The range of physical movements and emotional expressions exhibited by service robots is comparatively restricted (Kim et al., 2019).

Source: Walters et al. (2008), Belanche et al. (2019), and Kim et al. (2019).

Robot socialization refers to the integration of conventional interpersonal communication and emotional technologies into robotic systems, aimed at improving their communicative capabilities with humans. The extent of social interaction is achieved through an ongoing process of development and adaptation. A fundamental prerequisite for effective social interaction is the robot’s ability to adjust to various social contexts and to convey its comprehension of these contexts via screens, keyboards, or synthetic speech systems, thereby facilitating the necessary transmission of information. The robot may create the appearance of comprehension without possessing genuine understanding. This phenomenon is exemplified by the Wizard of Oz technique, which enables users to engage with an interface while remaining unaware that the responses are produced by a human operator who is programming the robot’s behaviors, rather than by the robot autonomously. Beyond mere emotional expression, this communicative function encompasses the construction and maintenance of intricate social models, as well as the demonstration of requisite competencies in complex social environments. Communication can manifest in various forms, predominantly within auditory and visual domains. For the purpose of socialization, robots are not required to convey information with the same complexity as humans; rather, they must possess adequate communicative abilities to interact with human users. Social robots should exhibit certain linguistic skills, gestural expressions, and emotional communication capabilities to foster perceptual expressiveness. This anthropomorphic quality enhances human understanding of the social competencies of robots, allowing them to be perceived as active participants within the human social sphere. The personification of robots can mitigate challenges related to human acceptance (Goetz et al., 2003; Martin et al., 2006) and is likely to be a critical factor in elucidating consumer responses during interactions with robotic systems (Kim et al., 2019). The presence of more human-like characteristics in robots, including appealing aesthetics and extroverted behaviors, facilitates greater acceptance and compliance from individuals (Goetz et al., 2003). Furthermore, customers may engage with robotic service providers in a manner akin to their interactions with human service providers (Hur et al., 2015; Kim et al., 2016).

The emergence of advanced anthropomorphic technology may elicit feelings of fear, bias, and potential discrimination against technologies that are perceived as embodying “speciesism” (Kim et al., 2019). For instance, the extensive deployment of anthropomorphic robots, particularly those that closely resemble humans yet remain artificial constructs, may provoke adverse reactions, including fear or rejection among individuals (Mori et al., 2012). The Uncanny Valley hypothesis remains relevant in this context, suggesting that consumers may experience apprehension toward robots that exhibit a high degree of human likeness (Mende et al., 2019). Although the specific triggers of these negative responses are not yet fully understood, existing research indicates that humanoid robots tend to provoke more reluctance and negative reactions compared to robots designed with pet-like or more utilitarian forms (Austermann et al., 2010). The underlying rationale for this phenomenon can be explained by the theory of the uncanny valley. According to this theory, the incorporation of human-like characteristics into robots enhances their acceptability and anthropomorphism to a certain extent. However, beyond a specific threshold of resemblance, an excessive likeness may paradoxically lead to a decrease in acceptability (Mori et al., 2012). Furthermore, highly humanoid robots may facilitate a greater understanding of mortality, thereby contributing to perceptions of these robots as mysterious and alien (Miriam et al., 2016).

## 5 The cognitive psychological mechanism underlying the phenomenon of robot anthropomorphism

The phenomenon of anthropomorphism can be fundamentally understood as a cognitive process wherein human attributes are ascribed to non-human entities. The psychological mechanisms that govern individuals’ perceptions and attentional focus toward others in their everyday experiences similarly influence their cognitive engagement with non-human subjects. Two prominent theoretical frameworks that have been proposed to elucidate the phenomenon of anthropomorphism in previous studies are the mere appearance hypothesis and the SEEK (sociality, effectance, and elicited agent knowledge) theory (Shepard, 1987; Zhao and Malle, 2022; Dubois-Sage et al., 2023). The SEEK theory was introduced by Epley et al. (2007). This framework operates collaboratively to modulate the degree to which individuals attribute human-like characteristics to nonhuman agents at any given moment. It achieves this by influencing the activation, correction, or application of anthropomorphic knowledge in relation to a specific target during the process of inductive reasoning. The activation of subject knowledge, effectiveness motivation, and social motivation is directly shaped by various factors, including personality traits, contextual elements, developmental stages, and cultural influences (Epley et al., 2007). Personality traits denote stable individual differences that can impact the sustained activation of specific knowledge representations or motivational states. Contextual factors pertain to the immediate characteristics of the environment, which can modify the accessibility of knowledge representations, thereby enhancing or diminishing effectiveness and social motivation. Furthermore, developmental and cultural factors play a significant role in shaping the expression of anthropomorphism by affecting the content of agent representations and the intensity of effective motivation across different developmental stages and cultural contexts. In the review performed by Dubois-Sage et al. (2023), contextual factors are classified as situational factors, whereas user-related factors encompass human elements, which consist of personality traits, developmental stages, and cultural influences. A thorough comprehension of the three psychological determinants--subject knowledge activation, effectiveness motivation, and social motivation--alongside their modulation by specific variables such as personality, context, development, and culture, is essential for a nuanced understanding of anthropomorphism in everyday life. Table 2 delineates the specific independent variables associated with these three factors that influence anthropomorphism.

**Table 2:** The origins of influence regarding the primary psychological factors contributing to anthropomorphism.

Independent variable category	Key psychological determinants		
	Elicited agent knowledge	Effectance motivation	Sociality motivation
Dispositional	Need for cognition	Need for closure, desire for control	Chronic loneliness
Situational	Perceived similarity	Anticipated interaction, apparent predictability	Social disconnection
Developmental	Acquisition of alternative theories	Attaining competence	Attachment
Cultural	Experience, norms, and ideologies	Uncertainty avoidance	Individualism and collectivism

Source: Epley et al. (2007).

### 5.1 Factors influencing the activation of agent knowledge

The phenomenon of anthropomorphism is significantly shaped by human cognitive factors (Epley et al., 2007). These cognitive factors influence the activation of knowledge related to human characteristics, whether derived from long-term memory or situational contexts, thereby modifying anthropomorphic understanding and facilitating the application of human knowledge to the behavior of robots during reasoning processes. In the context of cognitive robotics, individuals’ self-knowledge and general knowledge frequently serve as foundational elements for inductive reasoning. This is attributable to a critical phase in human cognitive development, which involves the ability to differentiate oneself from others, including the recognition of non-human agents (Gopnik and Meltzoff, 1994). To perceive others as distinct entities, it is essential to cultivate a more sophisticated self-concept and leverage this self-awareness to simulate the experiences of others, thereby inferring their psychological states (Meltzoff and Brooks, 2001). Furthermore, categorical knowledge regarding humans, often referred to as general knowledge, offers a nuanced and comprehensible cognitive framework that can be effectively employed in reasoning about robots (Inagaki and Hatano, 1987).

From a dispositional perspective, the activation of subject knowledge represents a fundamental cognitive necessity for human beings. The processes of self-cognition and its phenomenological aspects serve as the intuitive foundation for anthropomorphic reasoning in the context of robotic cognition. Variations in attentional resources among individuals can significantly influence their propensity for anthropomorphism toward robots. Individuals with heightened cognitive needs tend to engage in more profound analytical thinking, which enables them to mitigate biases in their judgments (Epley and Gilovich, 2010).

From a situational influence standpoint, the activation of subject knowledge facilitates the acquisition of perceptual similarities. When individuals perceive a resemblance between their goals and their own identity, they are more likely to draw upon self-referential knowledge when reasoning about others (Ames, 2004). The degree of anthropomorphism that individuals attribute to robots can be affected by the similarity of goals between humans and robots. Observable humanoid characteristics can shape the anthropomorphic knowledge framework, thereby increasing the likelihood of applying such knowledge to robotic entities (Mussweiler, 2003). In this context, morphological and motor similarities are particularly significant; the presence of human-like faces and bodies in robots and mechanical devices enhances the likelihood of anthropomorphic responses (DiSalvo et al., 2002). As technological advancements continue to progress, robots are increasingly poised to achieve greater levels of autonomy (Stapels and Eyssel, 2022). Robot autonomy is characterized as “the degree to which a robot is capable of perceiving its surroundings, formulating plans based on that information, and executing actions within that environment to achieve a specific task-oriented objective (whether predetermined or generated by the robot) without external intervention” (Beer et al., 2014).

From a developmental perspective, the activation of subject knowledge aids individuals in mastering the theory of alternation. Assessing the similarities between robots and humans, or between robots and the self, necessitates that evaluators possess prior knowledge of humans or the self, rather than relying solely on detailed descriptions of robots for inductive reasoning. Such representations are acquired through both direct and indirect learning experiences, which are influenced by various developmental factors. Development not only enhances individuals’ understanding of themselves and others but also shapes their cognitive frameworks regarding robots.

Finally, from a cultural influence perspective, the activation of subject knowledge serves as a foundational element for the formation of experiences, norms, and ideologies. The impact of culture parallels that of development, as it influences the anthropomorphism process by shaping the expressions of self, others, and non-human entities. When individuals possess fewer cognitive representations of artificial robots, the likelihood of activating their self-representations or general human representations upon encountering any robot increases, thereby facilitating the projection of human attributes onto the robotic entity. Consequently, culture plays a significant role in shaping the anthropomorphism process (Medin and Atran, 2004).

### 5.2 Sources of influence on motivation for effectiveness

Personification serves to enhance predictability and comprehension in an uncertain environment. In the absence of supplementary information, an individual’s self-awareness regarding their own preferences can facilitate others’ understanding of those preferences (Dawes and Mulford, 1996). Consequently, anthropomorphism is shaped by human motivation in the context of managing uncertainty, seeking meaning, and achieving effectiveness. Individuals exert control over their surroundings by increasing predictability and perceived controllability (Averill, 1973; Rothbaum et al., 1982). As a specific inductive process, anthropomorphism is motivated by the desire for effectiveness. A strong motivation for effectiveness correlates with a heightened degree of anthropomorphism, whereas a weak motivation corresponds to a diminished degree of anthropomorphism. Additionally, within the realm of robotics, the predictive capacity of effectiveness motivation can be influenced by two primary factors. First, the uncertainty associated with either real or hypothetical robot behavior can amplify anthropomorphism. This uncertainty stems from the novel and unfamiliar nature of robots, which may exhibit unpredictable behaviors, defy individual expectations, or involve causal mechanisms that are either unknown or unobservable. Second, anthropomorphism is linked to the motivation for accurately understanding or predicting robot behavior. When the motivation for precise understanding and accurate prediction is robust, the degree of anthropomorphism is likely to increase; conversely, when this motivation is weak, the degree of anthropomorphism is expected to decrease.

From a personality influence perspective, the anthropomorphism of robots addresses individuals’ intrinsic needs for closure and control. Those with a pronounced need for closure are more likely to engage with anthropomorphic representations. Consequently, individuals who exhibit a strong desire for closure may demonstrate a heightened inclination toward anthropomorphism. The desire for control pertains to the extent to which individuals are motivated to perceive themselves as governing the events in their lives (Burger, 1992). Individuals with a robust desire for control tend to engage in more pronounced attribution activities, often centered on typical anthropomorphic constructs such as intention and desire (Burger and Hemans, 1988).

From a situational influence standpoint, the anthropomorphism of robots significantly enhances the predictability of future interactions. The potential for future engagement should be regarded as a situational factor influencing anthropomorphism, as it shapes individuals’ expectations regarding future robotic behavior. This anthropomorphism also increases the likelihood of activating pre-existing anthropomorphic representations during interactions with robots. The additional information acquired while reasoning about robots may improve predictability and comprehension, thereby intensifying individuals’ anthropomorphic perceptions of these entities (Gilbert, 1998). Recent technological developments illustrate a significant enhancement in the autonomy of robots. Research indicates that robots are increasingly capable of navigating independently (Chen et al., 2019), making autonomous decisions regarding their interaction strategies (Senft et al., 2019), and engaging in increasingly meaningful conversations without human intervention (Chai et al., 2016).

From a developmental perspective, the anthropomorphism of robots plays a crucial role in enhancing human capabilities. As a mechanism for reducing uncertainty, anthropomorphism is particularly significant during the formative stages of an individual’s life. For individuals who have not yet fully acclimated to their environment, the activation of anthropomorphic representations to comprehend robots is relatively straightforward. Through the personification of robots, individuals gradually develop cognitive competencies related to these machines.

Finally, from a cultural influence perspective, the anthropomorphism of robots aids individuals in mitigating uncertainty. Uncertainty avoidance refers to the degree to which members of a culture feel threatened by ambiguous or unknown circumstances (Hofstede, 2001). By attributing human characteristics and behaviors to robots, anthropomorphism facilitates more familiar and predictable interactions between individuals and robots.

### 5.3 Sources of influence on social motivation

In the field of anthropomorphic research, the fulfillment of the need for social connections predominantly occurs through interactions with three prevalent non-human agents: pets, religious figures, and robots. Firstly, social motivation enhances the fundamental accessibility of social cues, which include humanoid characteristics and attributes (Gardner et al., 2005). This enhancement leads to an increased perceptual inclination toward humanoid features, a tendency that is also observed in robots. Secondly, social motivation further amplifies the propensity for personification of robots by encouraging individuals to actively seek out sources of social connection within their environment. Consequently, variations in social connections, influenced by factors such as personality, context, developmental stage, or cultural background, may impact the degree of anthropomorphism by modifying the accessibility of typical human traits. This influence can result in a diminished tendency to correct intuitive anthropomorphic inferences or an increased likelihood of attributing anthropomorphic characteristics to robots.

From a personality influence perspective, the anthropomorphism of robots can serve as a means to mitigate long-term loneliness in certain individuals. Those who have experienced prolonged feelings of loneliness often seek alternatives to traditional social interactions, such as companionship from pets, religious symbols, or robotic entities. For these individuals, the process of personification is more readily embraced. In contrast to individuals who maintain enduring social relationships, those who endure long-term loneliness exhibit a greater propensity to anthropomorphize robots.

From a situational influence standpoint, robot anthropomorphism can assist individuals in alleviating their feelings of social isolation. Individuals who suffer from the distress associated with isolation, exclusion, or separation from others frequently engage in efforts to mitigate their emotional pain by pursuing meaningful social connections (Maner et al., 2007). One effective strategy for these individuals to re-establish social connections is through the use of anthropomorphic robots, which can evoke human-like sensations in non-human contexts. Consequently, individuals who perceive themselves as disconnected from society are particularly inclined to anthropomorphize robots. Increased levels of robot autonomy are correlated with perceptions of enhanced robot intelligence (Choi et al., 2014), a decrease in user workload, improved user-friendliness, and greater adaptability (Stapels and Eyssel, 2022). Autonomous robots are associated with the mitigation of burdens by providing assistance in household tasks and other less desirable daily activities (Horstmann and Krämer, 2019).

From a developmental impact perspective, the anthropomorphism of machines can enhance individuals’ sense of attachment. Attachment is defined as the emotionally based bond that develops between an individual (such as an infant) and a specific object (such as a caregiver). This bond reflects the interplay between emotional connection and psychological processes (Bowlby, 1973). The nature of attachment influences the extent to which individuals actively seek information about social relationships. Individuals with insecure attachment styles are more likely to seek emotional fulfillment from non-human agents, such as robots (Epley et al., 2007).

Finally, from a cultural influence perspective, the anthropomorphism of machines illustrates the behavioral tendencies associated with individualism and collectivism. In collectivist cultures, there tends to be a higher overall level of social connections and support. Conversely, individualistic cultures typically exhibit a lower overall level of social connections and support. As a result, the dominant social motivations present in individualistic cultures may contribute to a more pronounced phenomenon of anthropomorphism.

## 6 Realistic considerations regarding the anthropomorphism of service robots

### 6.1 Expectations for service robots from a human perspective

The anthropomorphism of robots involves the integration of lifelike characteristics, which subsequently influences human expectations regarding the behavior and cognitive complexity of these machines (Duffy, 2003). The impetus for individuals to engage in social interactions with service robots primarily revolves around the objective of facilitating the execution of specific tasks or addressing human needs and requirements. This interaction fosters communication mechanisms between humans and robots (Kim et al., 2019). However, the endeavor to create human-like robots may inadvertently constrain their functional capabilities. It is important to recognize that the anatomical and functional attributes of humans should not serve as the definitive benchmark for robotic design, as robots fundamentally remain machines rather than human counterparts. This perspective does not pose a challenge to human identity; rather, it aims to enhance the efficacy of robots in fulfilling human needs. For a social robot to be deemed successful, it is not essential to obscure the distinctions between robots and humans. Instead, a judicious balance of anthropomorphic traits is necessary to convey that certain competencies can align with our expectations of socially intelligent entities (Stapels and Eyssel, 2022).

Social robots are increasingly recognized as the quintessential interface for human-computer interaction, necessitating the demonstration of seamless, coherent, and interactive social capabilities to embody the characteristics of machines. The convenience and efficiency of communication with robots, along with their functionality as assistants, are of paramount importance. This necessitates that robots acquire adequate authorization through the establishment of personality and identity traits, thereby enhancing human acceptance of robotic communication and social interaction mechanisms. It is essential to avoid presuming that robots must adhere to a specific operational framework, as such assumptions may impede their physical or social capabilities. Contrary to the prevalent notion that a human-like form serves as the ideal universal functional basis for robots, there exists an opportunity for robots to exhibit distinctiveness. Consequently, the design of robots should not be constrained to merely powerful humanoid functionalities and aesthetic considerations; rather, it should incorporate features that facilitate social interaction with humans as required.

From a pragmatic standpoint, robots ought to be conceptualized as machines that function by emulating human traits, including personality, gestures, facial expressions, and emotions, in order to enhance their societal roles. Consequently, social robots should align with human expectations of behavior rather than compel individuals to accept that robots possess human-like reasoning capabilities. The act of personification should not be regarded as a comprehensive solution to all challenges in human-computer interaction; instead, it necessitates further investigation to establish a “language” for such interactions. This language should serve to facilitate, rather than constrain, communication, as it encapsulates the fundamental principles and expectations that individuals adhere to within social contexts.

The endeavor of anthropomorphizing robots should extend beyond the mere creation of synthetic humans. The primary objective of anthropomorphism is to design systems that can effectively navigate our physical and social environments—such as utilizing tools, operating vehicles, and ascending stairs—while also serving as a means to foster social interaction among individuals. To mitigate prevalent misconceptions in human-computer interaction and to avoid neglecting the essential components of anthropomorphism, it is crucial to strike a balance between public expectations and the actual capabilities of machines. Currently, the resemblance between robots and humans in terms of physical appearance remains in its nascent stages, and further research is required to identify which humanoid features are most significant in facilitating social interaction.

### 6.2 The required level of intelligence for service robots

Huang and Rust (2018) posited that service work encompasses various categories of tasks, including mechanical, cognitive, and emotional tasks. They proposed a classification of artificial intelligence into distinct types based on the nature of these tasks. Specifically, mechanical artificial intelligence is suited for the execution and replacement of simple mechanical tasks. Analytical artificial intelligence is designed to manage and substitute tasks that involve information processing, logical reasoning, and mathematical computations. In scenarios where intuitive intelligence is required—such as in complex, creative, chaotic, and context-dependent tasks—this form of intelligence is capable of addressing and replacing those tasks. Lastly, empathetic intelligence is equipped to handle and replace tasks that necessitate social interaction, emotional awareness, the ability to recognize and comprehend the emotions of others, and the capacity to respond appropriately in emotional contexts. Mechanical intelligence and analytical intelligence are categorized as forms of weak artificial intelligence, whereas intuitive intelligence and empathetic intelligence are associated with strong artificial intelligence (Huang and Rust, 2018; Hollebeek et al., 2021).

Proponents of strong artificial intelligence assert that it is possible to replicate human intelligence within artificial systems (Noel and Tom, 2001; Adriana and Robert, 2017; Hollebeek et al., 2021). This perspective posits that the brain functions as a biological machine capable of interpretation and reproduction in an artificial context. Adherents of this mechanistic theory contend that a comprehensive understanding of the computational processes governing the brain’s characteristics and functions will elucidate human cognitive processes, thereby facilitating the development of artificial intelligence systems endowed with emotions and consciousness (Noel and Tom, 2001; Adriana and Robert, 2017; Hollebeek et al., 2021). Conversely, advocates of weak artificial intelligence argue that the term “artificial intelligence” inherently suggests that human intelligence can only be simulated (Hollebeek et al., 2021). From this viewpoint, artificial systems can merely create an illusion of intelligence, exhibiting behaviors that are associated with intelligent activity. The primary concern for proponents of weak artificial intelligence is not whether the system genuinely possesses intelligence, but rather whether it displays attributes that lead individuals to perceive it as intelligent. The essence of the Turing test lies in its ability to determine whether a machine can convince individuals that they are engaging in conversation with another human being, or at the very least, that they cannot discern it as merely a machine. It can be argued that the Turing test represents an overly simplistic assessment of intrinsic intelligence, allowing systems to employ clever strategies to lead individuals to conclude that machines possess intelligence. In the realm of social robotics, the effective application of anthropomorphism and cognitive capabilities will provide a framework for robots to assess their intelligence through conventional Turing tests.

### 6.3 Management of anthropomorphism in service robots

The effective management of anthropomorphism in service robots represents a critical consideration in their implementation. A significant factor in this context is the extent and characteristics of visual anthropomorphic features. Researchers in robotics have initiated investigations into the interactive dynamics between humans and robots through physical manifestations. Notable examples include the incorporation of expressive facial features, which underscore the significance of eye contact, alongside the integration of facial and eye tracking technologies. Furthermore, the use of visually iconic or highly realistic humanoid designs, such as synthetic skin and hair, is prevalent in conveying artificial emotional states. A particularly illustrative aspect is the design of robot heads, which reflects a certain degree of anthropomorphism in both their construction and functional capabilities. The objective of designing robot heads that resemble human models is to obscure distinctly “robotic” characteristics, thereby diminishing the communicative barriers between humans and machines. This approach effectively facilitates anthropomorphism and enhances social interaction between robots and humans, while simultaneously regulating the extent of anthropomorphic representation.

From an engineering standpoint, the attainment of pronounced anthropomorphic features poses considerable challenges. Engineering research is pivotal within the domain of artificial intelligence, particularly in robotics, where anthropomorphism is perceived as a more straightforward and rational approach to addressing artificial intelligence challenges through meticulous investigation. However, it raises the question of why other facets of artificial intelligence research necessitate such engineering endeavors. A comprehensive solution should encompass an integrative strategy that merges engineering methodologies with an exploration of fundamental artificial intelligence research issues. From the perspective of social robotics, it is pertinent to consider whether individuals would attribute cognitive capabilities to robots. In the context of social interactions with robots, individuals maintain specific social expectations. If these expectations can guide individuals in anthropomorphizing robots, leading to more successful social interactions and a reduction in frustrating experiences, then the process of anthropomorphism can be regarded as having a beneficial impact.

## 7 Conclusion

Regardless of the perspective adopted regarding the phenomenon of anthropomorphism in service robots, it is evident that these anthropomorphic entities are becoming increasingly embedded in our everyday lives. In addressing the challenges associated with anthropomorphism in service robots, it is crucial to foster the development of a distinct identity for these robots. Efforts should be directed toward creating an environment that facilitates the seamless integration of service robots into human society, allowing them to differentiate themselves within this social context through their unique identities. This differentiation enables individuals to construct social cognitive models of robots, thereby enhancing the perception of robots as socially competent participants. It is imperative to regard robots not merely as passive entities relegated to the periphery of social interactions, but rather as active participants capable of engaging naturally within social environments.

A critical consideration in the anthropomorphization of service robots is the enhancement of their autonomy. The degree of autonomy exhibited by social robots is shaped by their designated social roles, capabilities, and the expectations they hold for themselves and that others hold for them within particular social contexts. Autonomy necessitates that robots possess the capacity for independent interaction, supported by their inherent abilities and the surrounding social environment. An optimal autonomous system should strike a balance among familiarity (affinity), inspiration (iconic), and mechanical solutions (functionality). Collectively, these mechanisms aim to cultivate a certain level of artificial social competencies in robots, thereby fulfilling the criteria for artificial “life” and “intelligence.”

The World Health Organization has proposed that three fundamental factors should be taken into account and balanced during the design process of robots. First, it is essential to retain a specific number of robotic features to prevent users from misinterpreting the emotional capabilities of robots, thus ensuring the effective realization of their mechanical performance. Second, it is important to appropriately exhibit human-like characteristics to foster user comfort during interactions with robots. Lastly, conveying a rich array of product features is necessary to enhance user comfort in the utilization of robots (DiSalvo et al., 2002). We maintain a strong conviction that for robots and humans to achieve “success” across all dimensions, their coexistence must be grounded in reality.
